# Preferences of people living with HIV for features of tuberculosis preventive treatment regimens in Uganda: a discrete choice experiment

**DOI:** 10.1002/jia2.26390

**Published:** 2024-11-26

**Authors:** Hélène E. Aschmann, Allan Musinguzi, Jillian L. Kadota, Catherine Namale, Juliet Kakeeto, Jane Nakimuli, Lydia Akello, Fred Welishe, Anne Nakitende, Christopher Berger, David W. Dowdy, Adithya Cattamanchi, Fred C. Semitala, Andrew D. Kerkhoff

**Affiliations:** ^1^ Division of Pulmonary and Critical Care Medicine University of California San Francisco San Francisco California USA; ^2^ Department of Epidemiology and Biostatistics University of California San Francisco San Francisco California USA; ^3^ Center for Tuberculosis University of California San Francisco San Francisco California USA; ^4^ Infectious Diseases Research Collaboration Kampala Uganda; ^5^ Uganda Tuberculosis Implementation Research Consortium, Walimu Kampala Uganda; ^6^ Department of Epidemiology Johns Hopkins Bloomberg School of Public Health Baltimore Maryland USA; ^7^ Division of Pulmonary Diseases and Critical Care Medicine University of California Irvine Irvine California USA; ^8^ Department of Medicine Makerere University College of Health Sciences Kampala Uganda; ^9^ Makerere University Joint AIDS Program Kampala Uganda; ^10^ Division of HIV, Infectious Diseases, and Global Medicine University of California San Francisco San Francisco California USA

**Keywords:** TB, Uganda, values and preferences, latent tuberculosis infection, tuberculosis preventive treatment, person‐centred care

## Abstract

**Introduction:**

Tuberculosis (TB) preventive treatment (TPT) is recommended for people living with HIV (PLHIV) in high TB burden settings. While 6 months of daily isoniazid remains widely used, shorter regimens are now available. However, little is known about preferences of PLHIV for key features of TPT regimens.

**Methods:**

From July to November 2022, we conducted a discrete choice experiment among adult PLHIV engaged in care at an urban HIV clinic in Kampala, Uganda. Participants chose between two hypothetical TPT regimens with five different features (pills per dose, frequency, duration, need for adjusted antiretroviral therapy [ART] dosage and side effects), organized across nine random choice tasks. We analysed preferences using hierarchical Bayesian estimation, latent class analysis and willingness‐to‐trade simulations.

**Results:**

Of 400 PLHIV, 392 (median age 44, 72% female, 91% TPT‐experienced) had high‐quality choice task responses. Pills per dose was the most important attribute (relative importance 32.4%, 95% confidence interval [CI] 31.6–33.2), followed by frequency (20.5% [95% CI 19.7–21.3]), duration (19.5% [95% CI 18.6–20.5]) and need for ART dosage adjustment (18.2% [95% CI 17.2–19.2]). Latent class analysis identified three preference groups: one prioritized less frequent, weekly dosing (*N* = 222; 57%); another was averse to ART dosage adjustment (*N* = 107; 27%); and the last prioritized short regimens with fewer side effects (*N* = 63; 16%). All groups highly valued fewer pills per dose. Overall, participants were willing to accept a regimen of 2.8 months’ additional duration [95% CI: 2.4–3.2] to reduce pills per dose from five to one, 3.6 [95% CI 2.4–4.8] months for weekly rather than daily dosing and 2.2 [95% CI 1.3–3.0] months to avoid ART dosage adjustment.

**Conclusions:**

To align with preferences of PLHIV in Uganda, decision‐makers should prioritize the development and implementation of TPT regimens with fewer pills, less frequent dosing and no need for ART dosage adjustment, rather than focus primarily on duration of treatment.

## 1 INTRODUCTION

Tuberculosis (TB) preventive treatment (TPT) is strongly recommended to address the high disease burden among people living with HIV (PLHIV) in TB‐endemic settings [1]. Short‐course TPT regimens have been shown to be similarly effective and better tolerated than the conventional 6 or 9 months of daily isoniazid (6H or 9H) and are now recommended as options for TPT in updated World Health Organization (WHO) guidelines [1]. These regimens include 3HP (3 months of weekly isoniazid and rifapentine), 1HP (1 month of daily isoniazid and rifapentine), 3HR (3 months of daily isoniazid and rifampin) and 4R (4 months of daily rifampin). However, data on the preferences of PLHIV for key features that comprise and differentiate each of these regimens—such as treatment duration, frequency of dosing, number of pills per dose—are lacking.

Current WHO guidelines on TB preventive treatment were informed by a single preference study including only 10 participants living with HIV [2]. Preferences were assessed using Likert‐scale questions and participants rated all features evaluated as important (short duration, less frequent intake, fewer side‐effects, fewer clinic visits, fewer pills, no need for directly observed therapy and no need to change dosage of antiretroviral therapy [ART]). However, it remains unknown how PLHIV would value individual features, make trade‐offs between features, and ultimately choose between TPT regimens with different features. Such data are critical to inform decisions on scaling up TPT regimens and to guide future TPT regimen development.

Choice‐based preference elicitation methods, including discrete choice experiments (DCEs), are increasingly being utilized to more systematically characterize patients’ healthcare preferences and inform policy‐ and implementation‐related decisions [3, 4, 5, 6]. Compared to simple rating exercises such as Likert scale questions, DCEs measure trade‐offs through a series of repeated questions where participants must choose between two or more hypothetical alternatives (e.g. “Would you prefer option A or option B?”). Notably, DCEs have been shown to have good predictive value for health‐related choices [7], including for TPT regimens in a low TB burden setting [8].

We, therefore, conducted a DCE among adult PLHIV accessing routine HIV care in Kampala, Uganda. Our objectives were to (1) determine the relative importance of TPT regimen features; (2) simulate how willing participants were to trade one TPT feature for another; and (3) assess the heterogeneity in preferences and identify distinct subgroups of PLHIV with similar preferences.

## 2 METHODS

### 2.1 Setting and participants

We conducted a cross‐sectional survey that included a DCE from July to November 2022. The study took place at the Mulago Immune Suppression Syndrome (i.e. HIV/AIDS) clinic, in Kampala, Uganda. The clinic provides comprehensive HIV care to over 16,000 PLHIV and is the largest outpatient HIV clinic in the country.

Individuals were eligible for study participation if they were receiving HIV/AIDS care at the clinic, were 18 years or older, had not initiated a TPT regimen in the past year and were not currently receiving TB treatment. People who were unable or unwilling to provide informed consent or were currently in prisons and other closed settings were excluded. We defined the inclusion criteria to include individuals eligible for TPT. In Uganda, adult PLHIV with a negative TB symptom screen are eligible for TPT if they have been stable on ART for at least 3 months, and without any known contraindications to the available TPT regimens (3HP or 6H). Latent TB infection testing is not required and is uncommonly done. Although a repeat course of TPT is currently not recommended by the Ministry of Health, in practice TPT is offered every 2 years in the HIV programme.

### 2.2 Ethics, consents and procedures

The Makerere University School of Public Health Research and Ethics Committee, the University of San Francisco Institutional Review Board and the Uganda National Council for Science and Technology approved the study. All participants provided written informed consent.

### 2.3 DCE design

**Figure 1:**
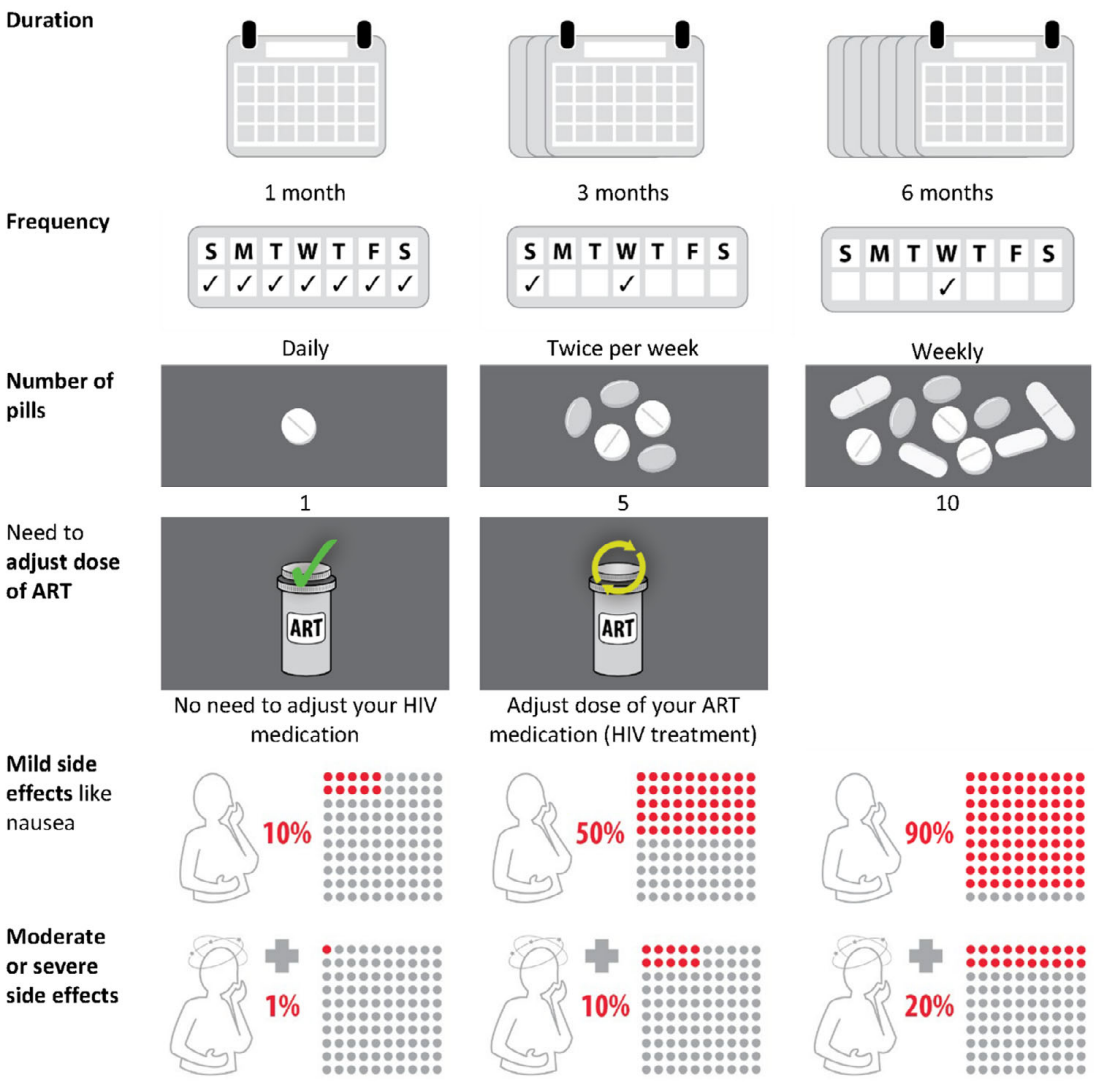
Attributes and levels in the discrete choice experiment describing different tuberculosis preventive treatment (TPT) regimens. This figure shows the final selection of attributes (column 1) and how levels were depicted to participants (columns 2–4). The need to adjust antiretroviral therapy (ART) dosage was described as requiring an increased dose, that is taking the ART twice daily during TPT, with an extra pill of the same ART drugs. Mild side effects were described as short‐lasting, such as nausea, dizziness, fatigue or skin rashes for a week or so, not requiring a stop of TPT or a clinic visit. Moderate or severe side effects were described as tingling of the feet, allergy or liver damage, requiring additional visits to the clinic and stopping TPT or being admitted to the hospital. ART, antiretroviral therapy; HIV, human immunodeficiency virus.

The DCE was designed using the “balanced overlap” method in Sawtooth Lighthouse Studio version 9.13.2 [9] to allow for analysis of interaction terms [10]. An initial list of DCE attributes and levels reflecting key features of TPT regimens was generated based on a review of the literature, refined by an interdisciplinary team, and further refined after pilot testing among 29 PLHIV. Pilot participants completed a preliminary version of the DCE and answered a short feedback survey (available upon request and as part of preprint [11]), where they were asked to explain attributes in their own words, describe the decision‐making process informing their choices, and provide feedback on the overall understandability, graphics and the length of the survey. As the final design differed from the preliminary designs, we did not include pilot participants in the final analysis. The final design included six attributes (duration of treatment in months, frequency of dosing, number of pills per dose, need to adjust the dose of ART, mild side effects and moderate or severe side effects) with 2–3 levels each (Figure 1). Each participant was randomly allocated to one of 500 randomly generated sets of nine random choice tasks. In addition, we included a dominant choice task to assess participant comprehension of the DCE (Table S1). All choice tasks were unlabelled (named “Treatment A” and “Treatment B”) and included all attributes that always appeared in the same relative position. Participants were first required to choose their preferred treatment (A or B), and then between their preferred treatment and no treatment (Figure S1).

### 2.4 Procedures

Potential participants were approached in the clinic's waiting area by an HIV peer counsellor. Specific recruitment efforts were undertaken to target persons who had never taken TPT and men. Participants received 20,000 Ugandan Shillings for their participation and were offered a snack. The survey was administered one‐on‐one by a trained interviewer using an electronic tablet. Interviewers first presented general information on TB prevention using a flipbook. Interviewers then explained the DCE attributes and levels using a flipbook with icons from the DCE (flipbook available upon request and as part of preprint [11]). In addition to the DCE component, the survey collected information on demographics (age, sex, education, occupation, distance to clinic), multidimensional poverty index [12] and medical history (height, weight, current medications) including history of TB (prior active TB disease and type, prior TPT, TPT completion, side effects on TPT) and HIV (HIV duration, ART duration and regimen, viral load).

### 2.5 Sample size

We considered the minimum sample size for the DCE to be 250 PLHIV based on the formula 500c/ta, where “c” is the product of the greatest number of levels for any two attributes, “t” is the number of choice tasks and “a” is the number of options per choice task [13]. To enable a pre‐specified subgroup analysis by sex, we targeted a sample size of 400 participants since a minimum of 200 participants per subgroup is recommended [13].

### 2.6 Statistical analysis

Analyses were performed in Lighthouse Studio version 9.13.2 by Sawtooth Software (hierarchical Bayes estimation, latent class analysis and willingness‐to‐trade simulations) and R version 4.1.2 (regressions and descriptive analyses). We excluded participants from analyses if: (1) the dominant fixed choice task was answered incorrectly, (2) the no treatment option was always selected in each of the nine random choice tasks, or (3) participants showed two or more signs of inattention or lack of understanding including “straight‐lining” (e.g. always choosing option A or option B), self‐reported difficulty understanding the tasks (“difficult” or “very difficult”) in a question at the end of the survey, and inconsistent choices indicated by a root‐likelihood (RLH) fit statistic below 0.651. The RLH threshold was selected based on simulations of random answers as described previously [14].

We used a hierarchical Bayesian model to calculate mean preference weights (also known as part‐worth utilities) for each attribute level (along with 95% CIs) and the relative importance of attributes (which add up to 100% across attributes). When estimating preference weights, each choice task was modelled as two independent choice tasks. The first task involved choosing between TPT options A or B (without a “none” option). The second task involved choosing among options A, B and the “none” option. If a participant indicated they would accept the TPT option they chose, then the first‐choice task was not included in the analysis due to redundancy.

We used latent class multinomial logit to identify groups of participants with distinct preferences. For the final model, the number of preference groups was selected by considering (1) which solution optimized statistical fit based on several information criteria, including Bayesian information criteria, which was the primary information criterion considered, (2) the interpretability of the distinct preferences represented by each group, and (3) the membership size of each group [15, 16, 17].

We implemented a Shares of Preference Model using the Sawtooth Choice Simulator tool to estimate participants’ willingness‐to trade treatment duration (in months) and number of pills per dose for other regimen features using 3HP (4 pills weekly for 3 months with 11.5% mild and 6.0% moderate or severe side effects) and 6H (2 pills daily for 6 months with 36.1% mild and 8.2% moderate or severe side effects) as competitors given their current availability as TPT options in Uganda. We defined moderate or severe side effects as requiring a clinic visit, and used reported adverse event rates for the simulations [18]. We calculated 95% CIs using 300 bootstrap samples and 30 competitive sets per sample, as recommended by Sawtooth Software [19]. We performed subgroup analyses according to preference groups identified from the latent class analysis.

## 3 RESULTS

### 3.1 Participant characteristics

**Figure 2:**
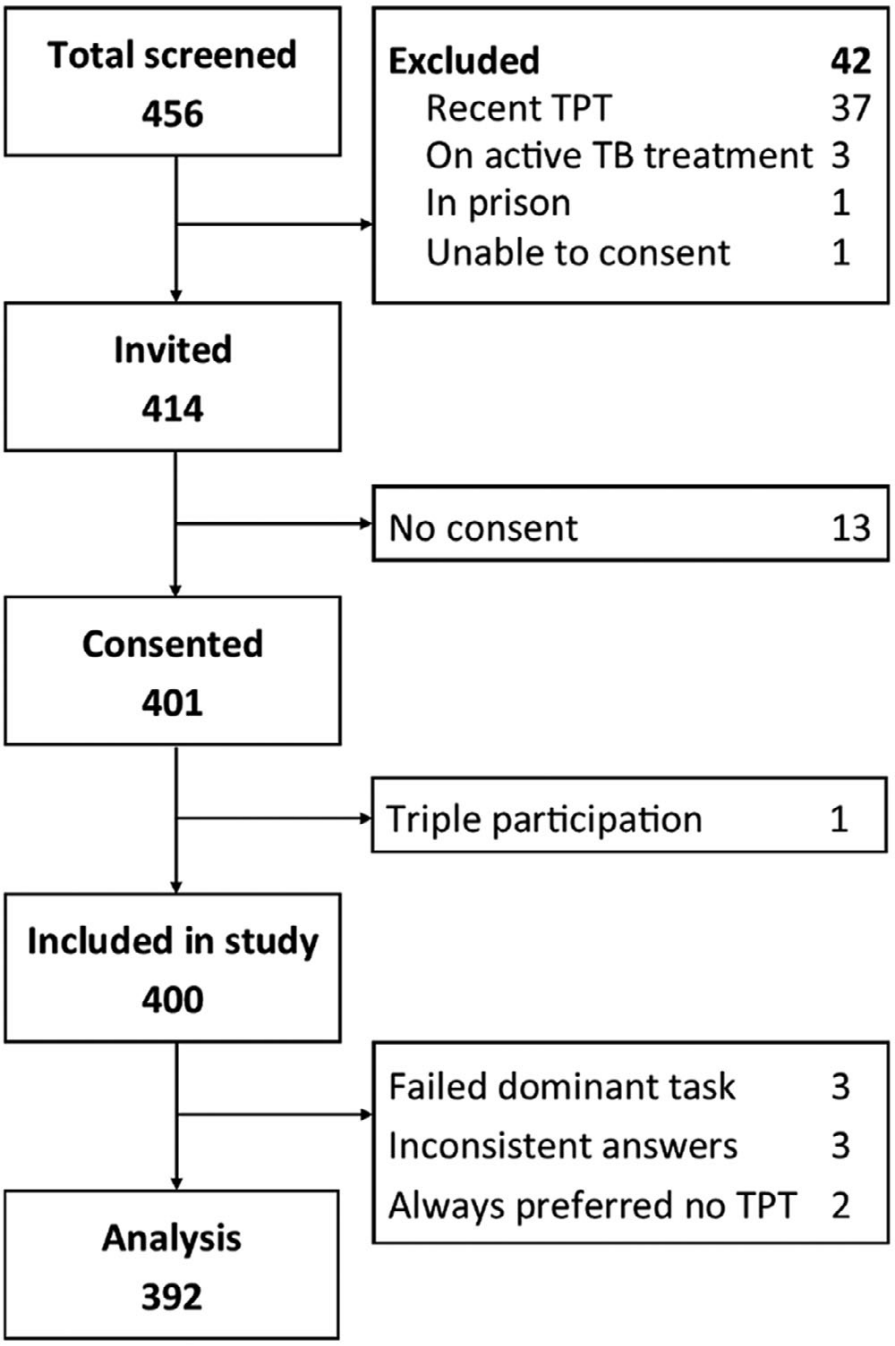
Participants’ study flow with 392 participants included in the final analysis. TB, tuberculosis; TPT, tuberculosis preventive treatment.

Of 456 persons screened, 414 were invited and 401 consented (response rate of 97% [401/414]) (Figure 2). We excluded one person from the study who was erroneously enrolled three times. Initial analysis excluded eight additional participants based on quality checks: three failed the dominant task, three had two signs of inattention or difficulty understanding choice tasks and two always selected the no treatment option. Across tasks, 60% (237/394) accepted all selected TPT regimens (i.e. never opted out), 39% (155/394) accepted some regimens and 0.5% (2/394) accepted none.

The majority of participants were female (72%), employed (80%) and ART‐experienced (100%), with a median of 10.4 years on ART (Table 1). Most participants had previously taken TPT (91%), with either 6H (68%) and/or 3HP (33%). Most participants found it easy or very easy to understand the DCE (88%) and to choose between TPT options in each DCE choice task (77%). Non‐participants (13 persons eligible and invited but who did not consent) were similar to participants with respect to age (mean 46.9, interquartile range [IQR]: 41−54) and sex (77% female).

**Table 1:** Participant characteristics at baseline, based on medical records and self‐report

Participants (*N* = 400)	*N* Median	(%) (IQR)
Female sex	288	(72%)
Age	44	(IQR: 38, 51)
BMI	26.1	(IQR: 22.2, 30.1)
Education		
None	91	(23%)
Primary	150	(38%)
Secondary	116	(29%)
Tertiary or higher	43	(11%)
Work status		
Hired	76	(19%)
Self‐employed	245	(61%)
Unemployed	45	(11%)
Not working	22	(6%)
Other	12	(3%)
Multidimensional poverty index^a^		
Severely poor	11	(3%)
Poor	63	(16%)
Vulnerable	111	(28%)
Prior TPT^b^		
Prior 6H	247	(68%)
Prior 3HP	121	(33%)
TPT completion (*N* = 365)		
TPT completed	353	(97%)
Do not know/do not want to answer	1	(0%)
Did you experience side effects from TPT? (*N* = 365)		
Yes, and I had to see my doctor about it.	28	(8%)
Yes, but only mild ones and I did not see my doctor about it.	58	(16%)
History of active tuberculosis	72	(18%)
Current antiretroviral therapy		
Dolutegravir‐based	378	(95%)
Efavirenz‐based	14	(4%)
Other	8	(2%)
Time on antiretroviral therapy (years)	10.4	(IQR: 7.2, 14.1)
Viral load		
Suppressed	394	(99%)
Unsuppressed (≥1000 copies)	3	(1%)
Not yet done, recent HIV diagnosis	1	(0%)
Missing	2	(1%)
Taking other medications^c^	147	(37%)
Herbal medicine use		
Within last month	90	(23%)
Within last year	98	(24%)
Longer than a year ago	128	(32%)
Hormonal contraceptives among women (*N* = 288)	52	(18%)

Abbreviations: 3HP, 3 months of isoniazid and rifapentine; 6H, 6 months of isoniazid; BMI, body mass index; HIV, human immunodeficiency virus; IQR, interquartile range; TPT, tuberculosis preventive therapy.

^a^ The multidimensional poverty index captures deprivations in health, education and living standards.

^b^ Three persons answered that they had both previously taken 6H and 3HP.

^c^ Currently taking other medications not including HIV medication or contraceptives.

### 3.2 Preferences for TPT regimen features

**Figure 3:**
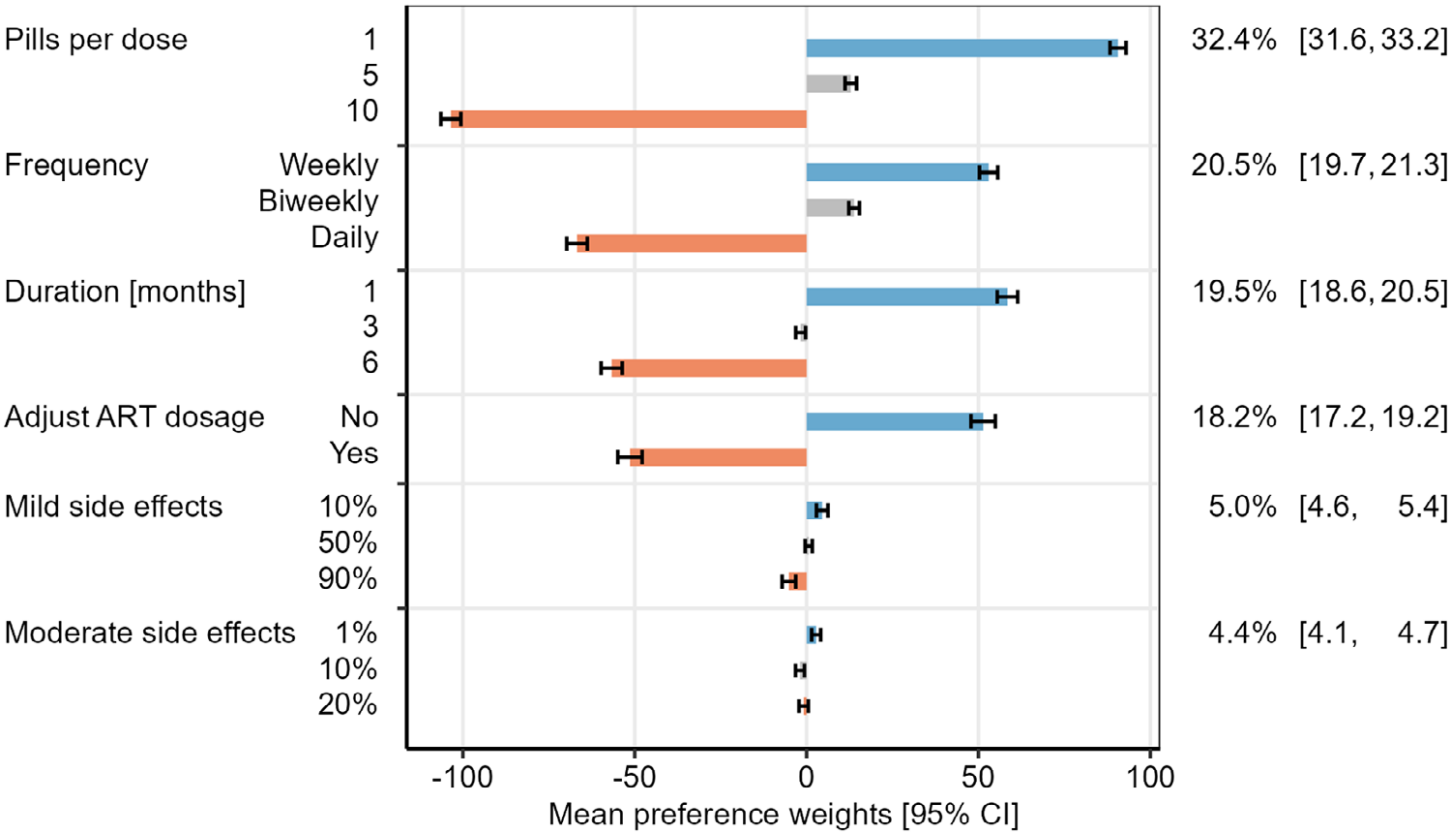
Mean preference weights of attribute levels and relative importance of attributes among all participants. Bars indicate the mean preference weights for each level among 392 participants using hierarchical Bayesian estimation. Blue bars indicate levels with the strongest positive preference (most preferred) per attribute, orange bars indicate levels with negative preference (least preferred). The percentage on the right side indicates the mean relative importance for each attribute. No treatment had a mean preference weight of −135.2 [95% CI −147.2, −123.2] (not shown). ART, antiretroviral therapy.

Overall, participants assigned the highest relative importance to the number of pills per dose (32.4% [95% CI 31.6–33.2]), with one pill per dose being strongly preferred compared to 10 pills per dose (Figure 3 and Table S2). Frequency of TPT dosing (relative importance 20.5% [95% CI 19.7–21.3]), duration of TPT (relative importance 19.5% [95% CI 18.6–20.5]) and need for ART dosage adjustment (relative importance 18.2% [95% CI 17.2–19.2]) were all similarly important. These relative importances were driven by strong preferences for weekly compared to daily or biweekly dosing, 1 month compared to either 3‐ or 6‐month durations, and no need for ART dosage adjustment compared to those requiring ART dose adjustment, respectively (Figure 3 and Table S2). Side effects were considered much less important than other attributes (relative importance 5.0% [95% CI 4.6–5.4] for mild side effects and 4.4% [95% CI 4.1–4.7] for moderate or severe side effects), with little difference in the relative value placed on 10% compared to a 50% or 90% frequency of mild side effects, or 1% compared to 10% or 20% frequency of moderate side effects. In a sensitivity analysis that included all 400 respondents, preference weights did not differ meaningfully compared to those of the 392 included in the primary analysis (Table S3). The same was true in a sensitivity analysis restricted to respondents with prior TPT experience only (*n* = 365, Table S4).

### 3.3 Heterogeneity of preferences for TPT features

**Figure 4:**
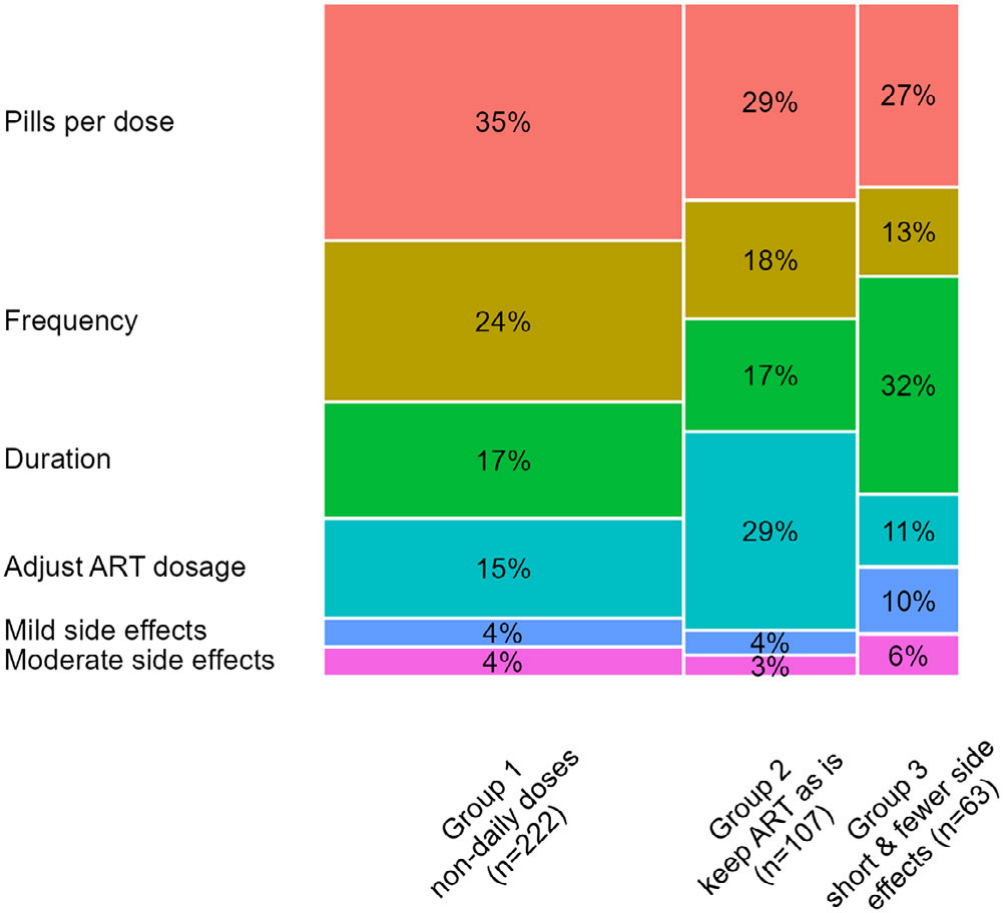
Mosaic plot showing the mean relative importance modelled using hierarchical Bayesian analysis among three groups identified by latent class analysis. The width of each column corresponds the proportion each group comprises of the overall population. ART, antiretroviral therapy.

Using latent class analysis, we identified three preference groups (Figure 4 and Figure S2), all of whom preferred fewer pills per dose and none of the groups prioritized avoiding moderate or severe side effects. Group 1, the largest group (*N* = 222, 57%, “non‐daily doses”), in addition to a strong preference for fewer doses, also prioritized less frequent dosing. This group had less strong preferences for a shorter TPT duration and avoidance of ART dosage adjustment. Group 2 (*N* = 107, 27%, “keep ART as is”) strongly preferred TPT regimens that required no ART dosage adjustment. This group had a strong preference for fewer pills per dose, but less strong preferences for less frequent dosing and a shorter duration. Finally, group 3 (*N* = 63, 16%, “short and fewer side effects”) had strong preferences for shorter regimens and was the only group to demonstrate somewhat strong preferences for regimens with a lower risk of side effects. This group had less strong preferences for less frequent dosing and avoidance of ART dosage adjustment.

We explored the association of baseline characteristics with individual preference weights. We found no association between sex, age, poverty, working status, prior history of TB, and years on ART with individual preference weights for duration, number of pills per dose, frequency of dosing and side effects (Table S5). Prior history of TPT (TPT without side effects, TPT with side effects, no TPT) was not associated with preference weights for side effects (Table S5). However, participants with less ART experience were more averse (i.e. had stronger negative preferences) to TPT regimens requiring ART dosage adjustments (preference weights were 1.09 [95% CI 0.18–2.01] larger per additional year since ART initiation, Table S5). In addition, participants taking other medications were more averse to ART dosage adjustments (preference weights were −7.70 [95% CI −15.3 to −0.06] smaller for those taking other medications, Table S5), and participants with any education were less averse to a high risk (90%) of mild side effects than participants with no education (preference weights were 5.61 [95% CI 0.77–10.4] larger for any education compared to no education, Table S5).

### 3.4 Willingness‐to‐trade for more preferred TPT regimen features

**Figure 5:**
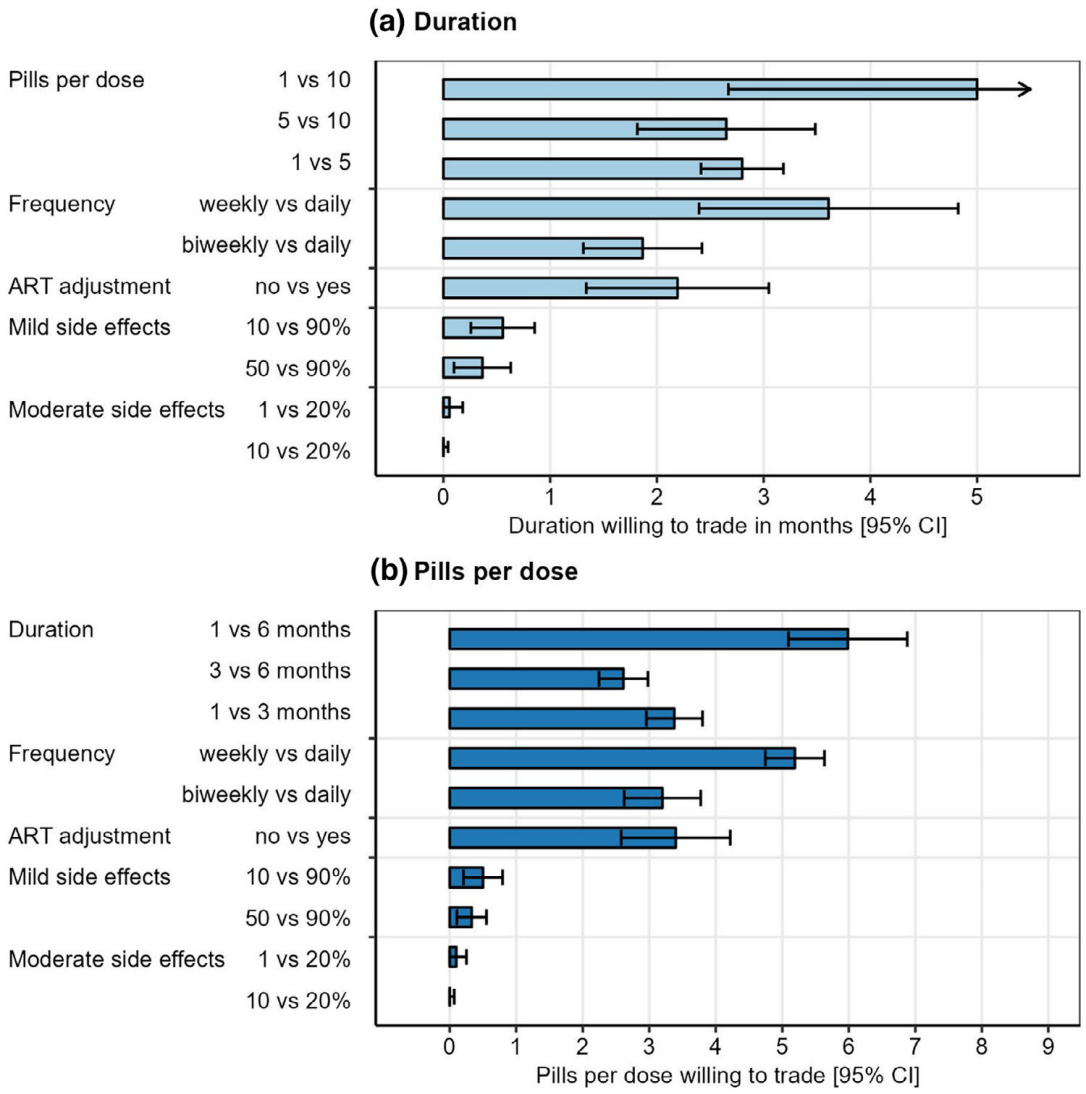
Willingness to trade (A) additional treatment duration months or (B) additional pills per dose for other improved regimen features. Results are truncated below zero months and above 5 months, and below zero pills and above 9 pills per dose (extrapolated values). The arrow in (A) indicates the upper confidence limit for 1 versus 10 pills was out of range. Antiretroviral therapy (ART) dosage adjustment was presented as requiring a second daily dose of ART. Moderate or severe side effects were described as side effects requiring medical care. ART, antiretroviral therapy.

We simulated trade‐offs between other regimen features and both treatment duration (in months, Figure 5A) and the number of pills per dose (Figure 5B). Overall (*n* = 392), participants were willing to take TPT for 2.7 [95% CI: 1.8–3.5] additional months in exchange for reducing the number of pills per dose from 10 to 5. If the number of pills per dose could be further reduced from 5 to 1, participants would have been willing to take TPT for another additional 2.8 [95% CI: 2.4–3.2] months. Participants were willing to take TPT for 3.6 [95% CI 2.4–4.8] additional months in exchange for weekly rather than daily dosing, and for 2.2 [95% CI 1.3–3.0] additional months in exchange for not needing ART dosage adjustment. Participants were willing to take TPT for only 0.6 [95% CI 0.3–0.9] additional months to reduce the risk of mild side effects from 90% to 10%, and were not willing to trade a longer duration of treatment for a lower risk of moderate or severe side effects. Similarly, participants were willing to take 3.4 [95% CI: 3.0–3.8] additional pills to reduce duration from 3 to 1 month, 5.2 [95% CI: 4.7–5.6] additional pills to reduce frequency of dosing from daily to weekly or 3.4 [95% CI: 2.6–4.2] additional pills in exchange for not needing ART dosage adjustment.

## 4 DISCUSSION

This DCE among adult PLHIV in Kampala, Uganda, provides important insights about what features of TPT regimens patients value the most. Although there was substantial heterogeneity of preferences as indicated by three distinct preference groups, all groups showed a very strong preference for lower pill burden. While TPT regimens as short as 1 month are now available, participants were willing to accept TPT regimens approximately 3 months longer in order to take 4 fewer pills per dose, 4 months longer to have weekly rather than daily dosing and 2 months longer to avoid ART dosage adjustment. Scale‐up of current regimens and future regimen development should consider pill burden, dosing frequency and compatibility with ART rather than focus exclusively on treatment duration.

Previous studies have also suggested that pill burden, dosing frequency and compatibility with ART are important considerations for TPT regimens. A study to characterize and understand gaps in the TPT care cascade among PLHIV in Uganda found that pill burden was an important barrier for patients [20]. We previously reported that 81% of PLHIV expressed a preference for 3HP over 1HP [21], supporting our finding here that less frequent dosing is preferred. Similarly, two previous studies focusing on paediatric TPT preferences in Eswatini and Peru found that less frequent dosing was valued [22, 23] even though daily dosing may be easier to remember [24]. Our DCE confirms weekly dosing is preferred among adults, too, and adds nuance by demonstrating how PLHIV make trade‐offs between these features and how trade‐offs differ between preference subgroups. The results of the latent class analysis show that a “one‐size fits all” approach is unlikely to be aligned with the wants of diverse individuals. These results suggest that the provision of TPT choice whenever possible—for example, shorter but more frequent dosing (1HP) versus longer but less frequent dosing (3HP)—is likely to maximize acceptability and uptake.

Notably, more than one‐in‐four participants (“keep ART as is” group) expressed a strong preference for maintaining their current ART regimen without adjustments. Participants with less ART experience were particularly averse to dosage changes. Additionally, those taking other medications unrelated to HIV were also more resistant to changes in their ART dosage, possibly due to concerns about potential drug‐drug interactions. Since 2019, the WHO has recommended dolutegravir‐based ART regimens as first‐line therapy for all PLHIV, and currently, over 20 million PLHIV globally are receiving these regimens [25]. While no adjustments to standard daily dolutegravir dosing are recommended for 3HP [26], preliminary data suggest that an adjustment to twice daily dolutegravir dosing is likely necessary for 1HP [27]. Our findings suggest that a significant subset of PLHIV may find the trade‐off of adjusting their ART to safely accommodate 1HP (as well as 3HR and 4H) unpalatable, potentially leading to decreased acceptance of TPT if only these regimens were offered.

Participants in our study generally placed a low value on avoiding mild and moderate or severe side effects compared to other TPT features, a finding that aligns with a best‐worst scaling choice exercise among PLHIV in South Africa [28]. We also found an association between higher education levels and a greater willingness to accept a high risk of mild side effects (90%), corroborating a qualitative study from South Africa that highlighted the role of education in shaping perceptions of TPT risks and benefits [29]. This underscores the importance of using culturally tailored, patient‐friendly educational materials in counselling, as we did prior to administering our DCE [30], to help especially those with lower health literacy grasp the trade‐offs involved in TPT acceptance. Our findings contrast with a DCE conducted among individuals with latent TB infection in Canada, where liver damage concerns related to TPT were prominent [31]. Differences in study populations, prior TPT experience, and TB risk may explain these opposing findings. For example, most of our participants (91%) had prior TPT experience, and only 24% reported experiencing any side effects. To ensure that our findings are immediately applicable to the current context in Uganda, we intentionally enrolled participants at the Mulago ISS clinic, a national centre of excellence in Uganda. Thus, we predominantly enrolled women living with HIV, consistent with the higher prevalence of HIV among women in Uganda, as well as people with prior TPT experience, reflecting the ongoing practice in Uganda and many countries of providing repeated TPT courses to PLHIV.

Our study had several strengths, including a large and representative sample of PLHIV in care in Kampala, Uganda, and the application of latent class analysis to uncover preference heterogeneity [32]. However, our study does have some limitations. First, due to resource limitations, we did not conduct a formal qualitative study to select attributes and refine them through full cognitive interviews. However, we used an iterative DCE design process with pilot testing using a structured feedback questionnaire that included open‐ended questions [33]. Second, most participants had taken ART for many years and had previously taken TPT, and it is possible that the preferences of people newly initiating ART may differ. Third, current TPT options, 1HP and 3HP as fixed‐dose combinations (FDCs), consist of 3−5 pills, including vitamin B6. We did not directly measure preferences for smaller differences in number of pills. Previously, these regimens consisted of up to 11 pills per dose [34], and we limited the number of levels to optimize statistical power and study feasibility. Fourth, while our findings offer valuable insights into the preferences of PLHIV in Uganda, further studies are needed to confirm the generalizability of these results to PLHIV in other countries. Although we did not assess how contextual factors influence preferences, socio‐political factors, such as Uganda's relative strength of healthcare infrastructure, widespread TPT availability and the criminalization of homosexuality, may affect the generalizability of our findings to settings with different socio‐political and healthcare landscapes. Finally, DCEs present hypothetical choices (“stated preferences”) that may differ from real‐world decisions (“revealed preferences”). However, their predictive value for actual health choices has been validated [8]. Moreover, they offer advantages over “revealed preferences,” which are limited to existing options and cannot predict the acceptability of future TPT regimens [29].

## 5 CONCLUSIONS

In conclusion, our study shows that while there are heterogeneous preferences for TPT‐related features among PLHIV in Uganda, there is a strong preference for regimens with lower pill burdens, less‐frequent dosing and no need for ART regimen adjustments. Most participants exhibited a willingness to undergo longer TPT regimens if they could access a TPT regimen with these preferred features. Collectively, our findings suggest that, in order to align with the preferences of PLHIV, policymakers should prioritize the implementation of FDC of existing TPT regimens and that future TPT regimens should prioritize reducing pill burden over further reducing treatment duration.

## COMPETING INTERESTS

The authors declare that they have no competing interests.

## AUTHORS’ CONTRIBUTIONS

HEA, AM, JLK, CB, AC and ADK conceptualized the study, HEA, AC and ADK led the methodology, HEA, DWD, AC, FCS and ADK secured funding. AM, CN, JK, JN, LA, FW and AN conducted the surveys, HEA, AM and JLK provided project administration, and HEA, AM, DWD, AC, FCS and ADK provided supervision. HEA and AM curated the data, HEA conducted the formal analysis, and HEA and AM validated the data analysis. HEA wrote the original manuscript draft, HEA, AC and ADK prepared the figures, and all authors participated in revising the manuscript and approved the final version.

## FUNDING

This study was supported by grants from the National Heart, Lung and Blood Institute (R01HL144406, AC and DWD) and National Institute of Allergy and Infectious Diseases (K23AI157914, ADK) of the National Institutes of Health, and by Early Postdoc.Mobility (191414, HEA) and Postdoc.Mobility (214129, HEA) fellowships from the Swiss National Science Foundation. HEA was also supported by the UCSF Center for Tuberculosis, NIH/NIAID P30: TB Research Advancement Center (UC TRAC) P30AI168440, and NIH/NIAID R25: TB Research and Mentorship Program (TB RAMP) 1R25AI147375.

## Supporting information



Supporting Information

## Data Availability

Deidentified individual participant data that support the findings of this study are available from the corresponding author upon reasonable request after approval of a proposal.
