# Supplementary material for: Preferences of people living with HIV for features of tuberculosis preventive treatment regimens in Uganda: a discrete choice experiment
Source: J Int AIDS Soc. 2024 Nov 26;27(12):e26390. doi: 10.1002/jia2.26390 (PMC11589386; doi:10.1002/jia2.26390)
Supplement: Supplementary file 1 — Supporting Information [file JIA2-27-e26390-s001.docx]

**Supplementary figures to: Preferences of people living with HIV for features of tuberculosis preventive treatment regimens in Uganda – a discrete choice experiment**

Hélène E. Aschmann, Allan Musinguzi, Jillian L. Kadota, Catherine Namale, Juliet Kakeeto, Jane Nakimuli, Lydia Akello, Fred Welishe, Anne Nakitende, Christopher Berger, David W. Dowdy, Adithya Cattamanchi, Fred C. Semitala, Andrew D. Kerkhoff

**Table S1**: Levels for dominant choice task

|  | Treatment A | Treatment B |
| --- | --- | --- |
| Duration | 6 months | 1 month |
| Frequency | Daily | Daily |
| Number of pills | 10 | 1 |
| Adjust ART dosage | No | No |
| Mild side effects | 90% | 10% |
| Moderate side effects | 20% | 1% |

**Table S2**: Hierarchical bayes mean utility (392 participants). This table shows the underlying values displayed in figure 3 in the main manuscript.

| **Attribute** | **Relative importance [95% CI]** | **Level** | **Mean preference weight**  **[95% CI]** | | |
| --- | --- | --- | --- | --- | --- |
| Number of pills | 32.4 [31.6, 33.2] | 1 | 90.6 | [88.3, | 92.9] |
|  |  | 5 | 12.9 | [11.2, | 14.5] |
|  |  | 10 | -103.5 | [-106.4, | -100.6] |
| Frequency | 20.5 [19.7, 21.3] | Weekly | 53.0 | [50.3, | 55.6] |
|  |  | Twice per week | 13.8 | [12.3, | 15.4] |
|  |  | Daily | -66.8 | [-69.8, | -63.8] |
| Duration (months) | 19.5 [18.6, 20.5] | 1 | 58.4 | [55.5, | 61.4] |
|  |  | 3 | -1.7 | [-3.2, | -0.3] |
|  |  | 6 | -56.7 | [-59.8, | -53.6] |
| ART adjustment | 18.2 [17.2, 19.2] | no | 51.4 | [47.8, | 54.9] |
|  |  | yes | -51.4 | [-54.9, | -47.8] |
| Mild side effects | 5.0 [4.6, 5.4] | 10% | 4.5 | [2.8, | 6.2] |
|  |  | 50% | 0.6 | [-0.4, | 1.6] |
|  |  | 90% | -5.1 | [-7.1, | -3.2] |
| Moderate side effects | 4.4 [4.1, 4.7] | 1% | 2.8 | [1.5, | 4.1] |
|  |  | 10% | -1.9 | [-3.2, | -0.6] |
|  |  | 20% | -0.9 | [-2.2, | 0.5] |
| No treatment | - | - | -135.2 | [-147.2, | -123.2] |

**Table S3**: Sensitivity analysis of hierarchical bayes mean utility that includes all 400 participants.

| **Attribute** | **Relative importance [95% CI]** | **Level** | **Mean preference weight**  **[95% CI]** | | |
| --- | --- | --- | --- | --- | --- |
| Number of pills | 32.2 [31.4, 33.0] | 1 | 89.9 | [87.5, | 92.4] |
|  |  | 5 | 12.4 | [10.8, | 13.9] |
|  |  | 10 | -102.3 | [-105.2, | -99.4] |
| Frequency | 20.4 [19.6, 21.1] | Weekly | 52.7 | [50.1, | 55.3] |
|  |  | Twice per week | 13.6 | [12.2, | 15.1] |
|  |  | Daily | -66.3 | [-69.2, | -63.4] |
| Duration (months) | 19.7 [18.8, 20.6] | 1 | 58.9 | [56.0, | 61.7] |
|  |  | 3 | -1.3 | [-2.8, | 0.2] |
|  |  | 6 | -57.6 | [-60.4, | -54.7] |
| ART adjustment | 18.4 [17.4, 19.4] | no | 51.5 | [48.0, | 55.1] |
|  |  | yes | -51.5 | [-55.1, | -48] |
| Mild side effects | 4.9 [4.5, 5.3] | 10% | 4.4 | [2.7, | 6.1] |
|  |  | 50% | 0.3 | [-0.7, | 1.4] |
|  |  | 90% | -4.7 | [-6.6, | -2.8] |
| Moderate side effects | 4.4 [4.2, 4.7] | 1% | 3.6 | [2.2, | 4.9] |
|  |  | 10% | -0.8 | [-2.1, | 0.4] |
|  |  | 20% | -2.7 | [-4.1, | -1.3] |
| No treatment | - | - | -145.7 | [-158.9, | -132.5] |

**Table S4**: Sensitivity analysis of hierarchical bayes mean utility restricted to participants with prior TPT experience (n=365).

| **Attribute** | **Relative importance [95% CI]** | **Level** | **Mean preference weight**  **[95% CI]** | | |
| --- | --- | --- | --- | --- | --- |
| Number of pills | 32.2 [31.4, 33.0] | 1 | 90.6 | [88.2, | 93.0] |
|  |  | 5 | 10.9 | [9.3, | 12.4] |
|  |  | 10 | -101.5 | [-104.4, | -98.6] |
| Frequency | 19.7 [19.0, 20.5] | Weekly | 50.1 | [47.3, | 52.9] |
|  |  | Twice per week | 14.1 | [12.2, | 15.9] |
|  |  | Daily | -64.2 | [-67.1, | -61.3] |
| Duration (months) | 19.9 [19.0, 20.8] | 1 | 59.5 | [56.6, | 62.4] |
|  |  | 3 | -1.4 | [-3.0, | 0.1] |
|  |  | 6 | -58.1 | [-60.9, | -55.2] |
| ART adjustment | 18.5 [17.4, 19.5] | no | 51.5 | [47.7, | 55.3] |
|  |  | yes | -51.5 | [-55.3, | -47.7] |
| Mild side effects | 5.3 [4.8, 5.7] | 10% | 3.8 | [1.9, | 5.7] |
|  |  | 50% | -1.7 | [-2.8, | -0.6] |
|  |  | 90% | -2.1 | [-4.2, | -0.1] |
| Moderate side effects | 4.5 [4.1, 4.8] | 1% | 1.4 | [-0.1, | 3.0] |
|  |  | 10% | -0.2 | [-1.5, | 1.2] |
|  |  | 20% | -1.2 | [-2.7, | 0.2] |
| No treatment | - | - | -143.3 | [-156.8, | -129.9] |

**Table S5**: Linear regression of preference weights for undesirable levels by participant characteristics based on hierarchical Bayesian estimation of individual preference weight. Negative values are less desirable, whereas positive are more desirable. For each undesirable level, the relative risk is given with the 95% confidence interval in brackets. Statistically significant effects at a level of 0.05 are highlighted in bold (not adjusted for multiplicity). Participants with education (compared to none) were less averse to mild side effects. Participants with longer duration since ART initiation were less averse to adjusting the ART dose, whereas participants who were taking other medications (for other conditions) were more averse to adjusting the ART dose.

|  | Duration: 6 months | | Number: 10 pills | | Frequency: daily | | ART: adjust dosage | | Mild side effects: 90% | | Moderate side effects: 20% | |
| --- | --- | --- | --- | --- | --- | --- | --- | --- | --- | --- | --- | --- |
| Intercept | -48.4 | (-63.9, -32.9) | -102.5 | (-117.1, -87.9) | -79.3 | (-94.4, -64.3) | -47.3 | (-66.5, -28.1) | -10.7 | (-20.7, -0.72) | 5.06 | (-1.75, 11.9) |
| Female vs male | 0.80 | (-6.40, 8.01) | 3.41 | (-3.40, 10.2) | 1.31 | (-5.70, 8.32) | -7.66 | (-16.2, 0.89) | 3.06 | (-1.61, 7.73) | -2.17 | (-5.36, 1.02) |
| Age per 10 years (ref: 18) | -0.76 | (-4.52, 2.99) | -0.06 | (-3.61, 3.49) | 3.38 | (-0.27, 7.03) | -1.52 | (-6.00, 2.97) | -0.39 | (-2.82, 2.05) | -0.19 | (-1.85, 1.48) |
| Education: any vs none | -0.74 | (-8.19, 6.72) | -4.14 | (-11.2, 2.91) | 1.64 | (-5.61, 8.89) | -8.19 | (-16.9, 0.49) | **5.61** | **(0.77, 10.4)** | -2.29 | (-5.59, 1.01) |
| Poor vs not poor^1^ | 5.16 | (-2.98, 13.3) | 1.65 | (-6.04, 9.34) | -2.64 | (-10.6, 5.28) | -3.83 | (-13.1, 5.44) | 1.66 | (-3.59, 6.92) | -0.25 | (-3.84, 3.34) |
| Working vs unemployed/other^2^ | -1.82 | (-9.72, 6.08) | 4.28 | (-3.19, 11.7) | 2.71 | (-4.97, 10.4) | 0.05 | (-8.92, 9.03) | -0.62 | (-5.74, 4.49) | -1.56 | (-5.05, 1.93) |
| Prior history of TB vs none | 5.47 | (-2.75, 13.7) | 4.66 | (-3.11, 12.4) | -7.87 | (-15.9, 0.13) | -8.55 | (-17.9, 0.80) | 3.23 | (-2.15, 8.62) | -2.42 | (-6.09, 1.26) |
| Years on ART (ref: 0) | -0.65 | (-1.46, 0.15) | -0.45 | (-1.21, 0.31) | 0.11 | (-0.67, 0.89) | **1.09** | **(0.18, 2.01)** | 0.07 | (-0.45, 0.59) | 0.03 | (-0.33, 0.38) |
| Prior TPT (ref: without side effects) |  |  |  |  |  |  |  |  |  |  |  |  |
| Never took TPT |  |  |  |  |  |  |  |  | -3.45 | (-10.9, 4.01) | -2.46 | (-7.56, 2.63) |
| Prior TPT with side effects |  |  |  |  |  |  |  |  | -3.67 | (-8.65, 1.31) | -1.87 | (-5.27, 1.54) |
| Other medications vs none^3^ |  |  |  |  |  |  | **-7.70** | **(-15.3, -0.06)** |  |  |  |  |
| Contraceptive use vs none^4^ |  |  |  |  |  |  | 3.12 | (-8.07, 14.3) |  |  |  |  |
| Herbal medicine use vs never |  |  |  |  |  |  | 6.45 | (-2.18, 15.1) |  |  |  |  |

^1^ Multidimensionally poor or severely poor vs not vulnerable or vulnerable. ^2^ Working includes hired or self-employed, unemployed/other includes unemployed, not working, and other. ^3^ Other medications than ART or contraceptives. ^4^ For contraceptive use, men were coded as non-users. ART: Antiretroviral therapy, TB: active tuberculosis, TPT: tuberculosis preventive treatment.


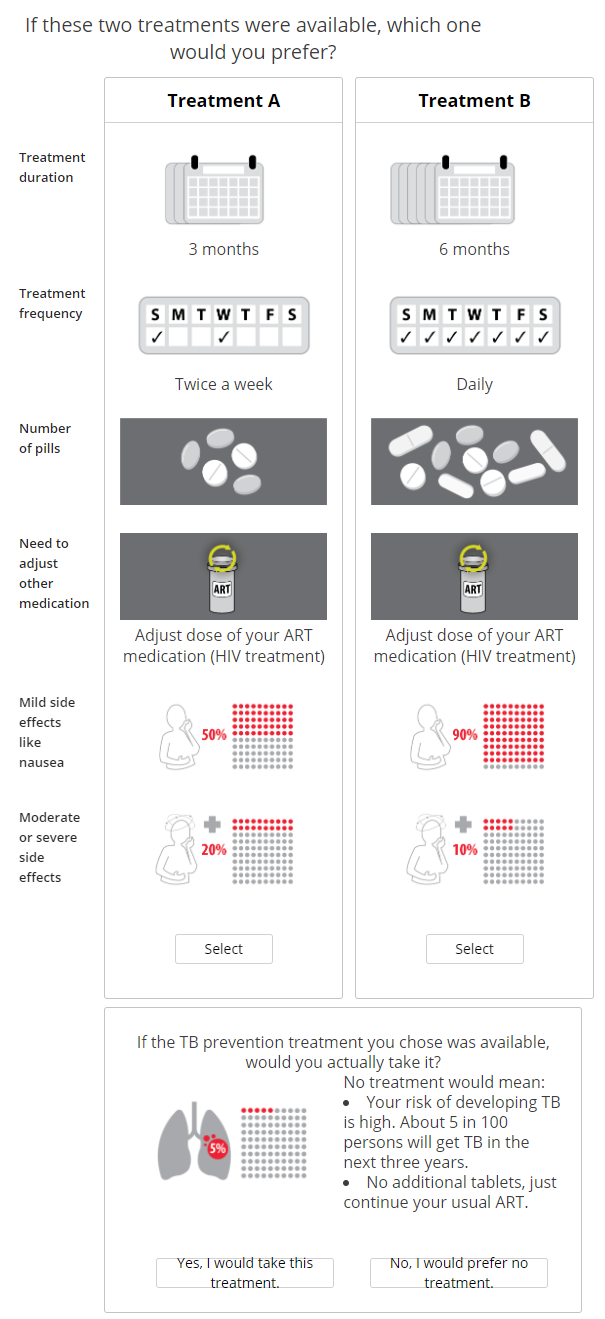


**Figure S1**: Example DCE random choice task (screenshot). The DCE was administrated on an electronic tablet. Participants were able to view all attributes and levels at once.

**
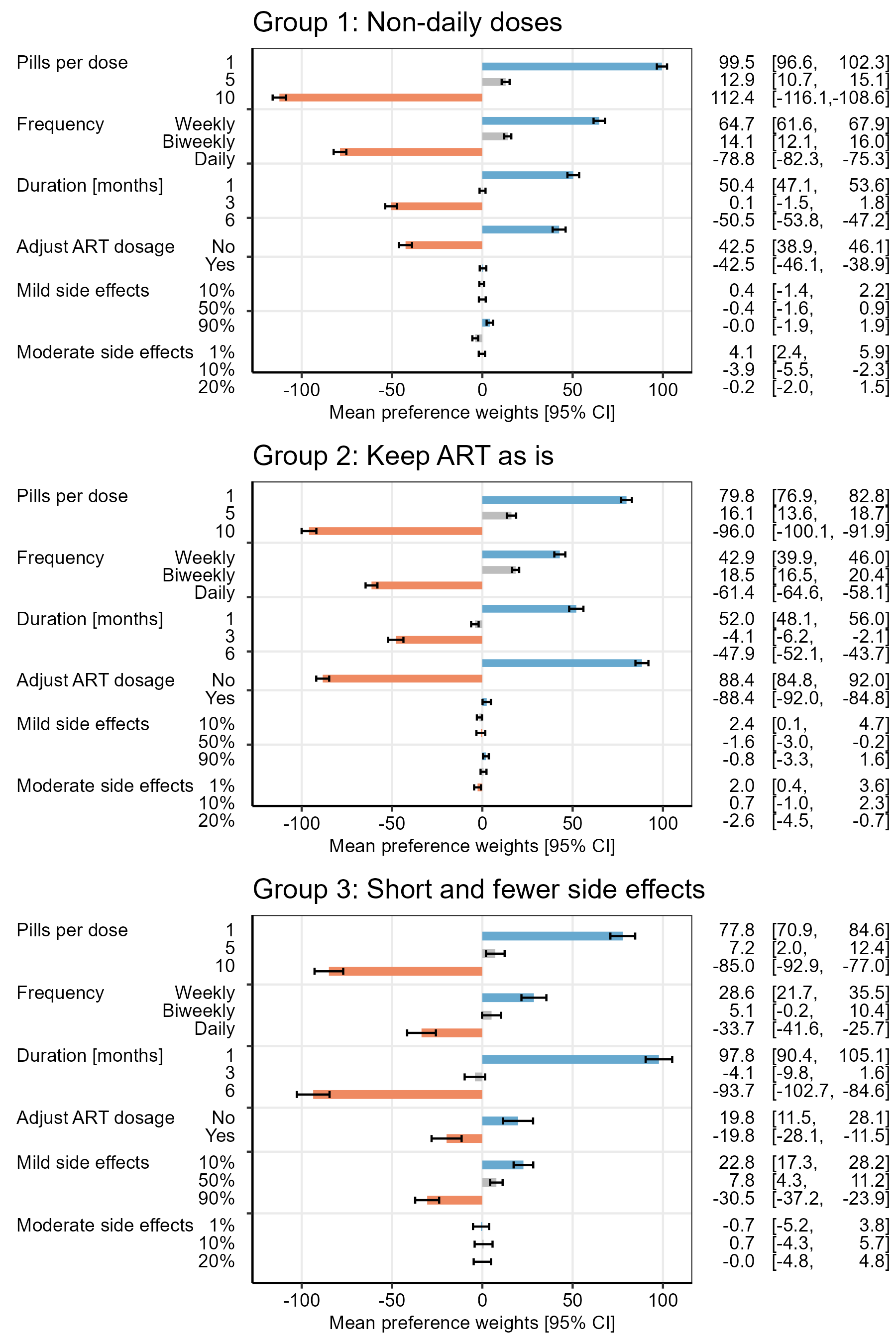
**

**Figure S2:** Mean preference weights by latent class [95% confidence interval]. Preference weights were estimated using hierarchical Bayes.
